# Host-microbiome interactions: Gut-Liver axis and its connection with other organs

**DOI:** 10.1038/s41522-022-00352-6

**Published:** 2022-11-01

**Authors:** Swadha Anand, Sharmila S. Mande

**Affiliations:** TCS Research, Tata Consultancy Services Ltd, Pune, 411 013 India

**Keywords:** Microbiome, Cellular microbiology

## Abstract

An understanding of connections between gut microbiome and liver has provided important insights into the pathophysiology of liver diseases. Since gut microbial dysbiosis increases gut permeability, the metabolites biosynthesized by them can reach the liver through portal circulation and affect hepatic immunity and inflammation. The immune cells activated by these metabolites can also reach liver through lymphatic circulation. Liver influences immunity and metabolism in multiple organs in the body, including gut. It releases bile acids and other metabolites into biliary tract from where they enter the systemic circulation. In this review, the bidirectional communication between the gut and the liver and the molecular cross talk between the host and the microbiome has been discussed. This review also provides details into the intricate level of communication and the role of microbiome in Gut-Liver-Brain, Gut-Liver-Kidney, Gut-Liver-Lung, and Gut-Liver-Heart axes. These observations indicate a complex network of interactions between host organs influenced by gut microbiome.

## Introduction

The role of gut microbial community (microbiome) in maintaining host health has gained a lot of attention in the recent past^1^. Scientific studies indicate links between dysbiosis or disturbance in microbiome and diseases that not only affect the gut, but also organs like brain, liver, lung, kidneys, etc. The pathophysiology of diseases effecting distal organs has often been associated with gastrointestinal discomfort or disorders. The crosstalk between the gut microbiome and distal organs is being increasingly recognized and host-microbiome interactions are being delineated piece by piece^2,3^. The increasing socio-economic burden of various diseases associated with changes in gut microbiome suggest the importance of understanding the molecular events facilitating such interactions.

Diseases affecting distal organ like liver (e.g., non-alcoholic/alcoholic fatty liver, non-alcoholic/alcoholic steatohepatitis, liver fibrosis/cirrhosis) are often seen to be associated with dysbiosis in gut microbiome^4–6^. Increase in fat cells in liver leads to a state known as fatty liver disease. Fatty liver may occur due to excessive intake of alcohol termed as ‘alcoholic fatty liver’ or may also be observed in individuals with no or negligible intake of alcohol termed as non-alcoholic fatty liver^7^. In both cases the observed pathological spectra may range from simple hepatic steatosis, steatohepatitis to liver cirrhosis^7^. In cases where an accumulation of fat leads to inflammation and damage, an advanced form of non-alcoholic fatty liver disease (NAFLD) called as Non-alcoholic steatohepatitis (NASH) is observed. Similarly, alcoholic steatohepatitis is the inflammatory state of Alcoholic liver disease (ALD)^8^. Since, these liver associated diseases are linked with dysbiosis in gut microbiome, it is important to understand the mechanisms involved in the cross-talk between gut and liver.

**Fig. 1:**
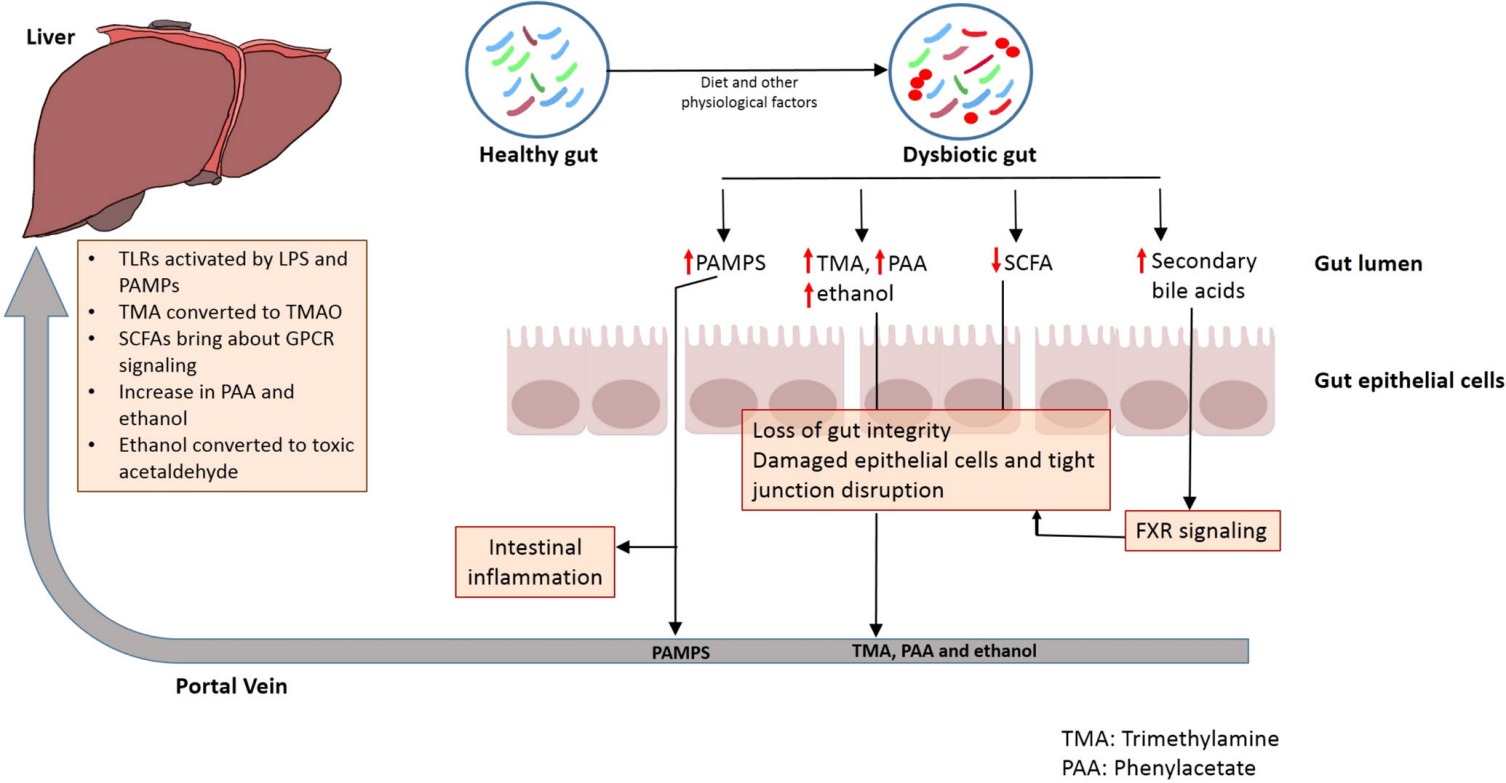
Translocation of metabolites in Gut–liver axis. Translocation of metabolites and pathogen associated molecular patterns (PAMPs) biosynthesized by microbes in the gut through the portal circulation to liver where they exert multiple effects on liver health condition.

Certain mechanisms govern the tight bidirectional communication between the gut and liver (Fig. 1). For example, metabolic machinery of the host and resident gut microbiome metabolize several exogenous dietary and environmental components as well as endogenous substrates like amino acids and bile acid. The products generated during this process are carried to liver by portal vein, thereby influencing hepatic physiology^9^. Similarly, the immune cells activated by several dietary compounds as well as metabolites from gut microbiome can enter lymphatic system and modulate immune responses in distal organ like the liver^9^. On the other hand, the liver communicates with the gut through the release of bile acids and other metabolites into biliary tract of the systemic circulation^9^. The release of bile salts by liver also helps to control unrestricted gut microbial growth^9^.

Liver dysfunction influences immunity and metabolism of not only the gut, but also other organs. For example, brain malfunction due to Hepatic encephalopathy (HE) as well as kidney disorders are often observed in people with liver ailments^10^. Hepatic encephalopathy (HE) leads to an impaired brain function often observed in patients with advanced liver diseases. The factors like decreased metabolism of ammonia associated with liver failure have often been associated with occurrence of HE^11^. Similarly, cardiovascular diseases effecting heart and blood vessels (CVD) are seen to be associated with fatty liver and other liver disorders^12^. Thus, it is important to understand the mechanisms involved in the interaction of Gut–Liver axis with distal organs. The beneficial effects of certain probiotics and Fecal Microbial Transfer (FMT) in liver diseases as well as ailments (like HE and CVDs) which are linked to Gut-Liver interaction with other organs further highlight the importance of the close-knit interaction of gut microbiome with host physiology.

### Gut microbiome and liver diseases

‘Non-alcoholic Fatty Liver Disease’ (NAFLD) and ‘Non-alcoholic Steatohepatitis’ (NASH) are liver ailments that have been associated with dysbiosis in gut microbiome and ‘Small Intestinal Bacterial Overgrowth’ (SIBO)^13,14^. Similar symptoms have also been observed in individuals suffering from ‘Alcoholic Liver Disease’ (ALD) due to excessive abuse of alcohol. The non- progressive form of these diseases (e.g. NAFLD) often involves fat accumulation in liver or steatosis, while the progressive form (e.g. NASH) is diagnosed by liver injury and inflammation (steatohepatitis)^9^. Analysis of stool samples of 57 patients showed lower levels of *Prevotella* and higher *Bacteroides* as well as *Ruminococcus* are seen in the gut of patients with NASH at stage 2 fibrosis or higher as compared to those in control subjects with fibrosis stage 1^15^. This indicates that gut microbiome changes are associated with severity of disease (fibrosis stage 1 vs. stage 2 in this case). Whole genome sequencing of gut microbial community obtained using stool samples indicates higher abundances of *Escherichia coli* and *Bacteroides vulgatus* in the gut of NAFLD patients in early (72 patients) as well as advanced stages of fibrosis (14 patients)^16^. Similarly, pediatric subjects suffering with NASH are seen to have higher occurrence of genus *Escherichia* as compared to obese non-NASH subjects^9^. Multiple studies on gut samples (stool as well as biopsy) of human as well as animal systems have indicated that while patients with ALD have an increased number of bacteria belonging to the family *Enterobacteriaceae* in their gut, they have lower abundances of genera *Lactobacillus* and *Bacteroidetes*^17,18^. Alterations in gut microbial community have also been observed in cirrhosis patients. Interestingly, analysis of stool samples of 95 liver cirrhosis patients and 47 healthy controls indicated an invasion of oral microbes like *Streptococcus* and *Veillonella* into the small intestine is observed in cirrhosis patients^19,20^. Bacterial genera like *Veillonella, Megasphaera, Dialister, Atopobium*, and *Prevotella* are also found in higher abundances in biopsies of distal duodenum from 30 cirrhosis patients as compared to 28 healthy controls used in the study^21^.

In addition to ascertaining the role of gut microbiome in liver diseases certain studies have indicated potential use of probiotics as a therapy for chronic liver diseases. The outcomes of these therapeutic strategies differ in terms of their efficacy and long-term impacts as well as effects on host-microbiome balance have yet to be elucidated. some of these studies have been mentioned in Supplementary Table 1. While most of these studies indicate a decrease in pro-inflammatory markers like cytokines, Lipopolysaccharides (LPS) etc. as well as improvement in liver lesions, few studies also indicate that no significant change is observed with the intake of probiotics in liver disease (Supplementary Table 1). The efficacy of a synbiotic (combination of a prebiotic (fructo-oligosaccharides and a probiotic *Bifidobacterium animalis subsp*. *lactis BB-12*) administered for 10–14 months on the gut microbiome, liver fat and fibrosis was studied as a part of a placebo controlled study called Insyte. The study recruited 55 NAFLD patients with symbiotic administration while 49 were administered a placebo. The results indicated that although a gut microbiome change was observed, no significant changes in liver pathophysiology could be observed^22^. Thus, long-term randomized controlled trials with larger number of participants are needed to clearly understand the efficacy of probiotics/prebiotics or symbiotics in amelioration of liver disease.

### Gut–Liver axis

#### Mechanisms of communication

##### Intestinal barrier integrity

The intestinal barrier, comprising of tightly bound cells, ensures selective transfer of nutrients and restricts the movement of pathogenic organisms from the gut lumen into the host system^23^. The gut microbiome influences gut barrier integrity by either maintaining immune signaling mechanisms or by producing metabolites like short chain fatty acids (SCFAs)^23^. Thus, disturbances in any of these factors can lead to an increase in gut permeability. For example, dysbiosis in gut microbiome in cases of inflammatory diseases or due to intake of high fat diet, alcohol and antibiotics can bring about loss of gut barrier integrity^24,25^.

A compromised gut barrier integrity is likely to lead to translocation of microorganisms and microbes- derived molecules into the portal system^26^. Under such condition, these microbes as well as their biosynthesized metabolites can translocate to the liver from where they can be carried through the portal system to distal organs, thereby causing their inflammation and injury (Fig. 1)^26^. Certain metabolites formed in the intestine may also directly interact with host factors in order to bring about exacerbation of liver disease^27,28^.

##### Transfer of microbes and microbes-derived metabolites through portal circulation

The intestinal dysbiosis is accompanied by loss of gut barrier integrity and transfer of pathogen associated molecular patterns (PAMPS) to the portal circulation^29^ (Fig. 1). This leads to induction of pattern recognition receptors (PRR) like TOLL-like receptors and NOD-like receptors in liver cells, which results in activation of pro-inflammatory signaling cascades, which in turn bring about local inflammatory responses^29^. Toll-like receptors are one category of PRRs which are suppressed in healthy liver conditions^30^ (Fig. 1). The delivery of pathogens or/and the molecules biosynthesized by them to the liver leads to activation of toll-like receptor (TLR) signaling. This leads to an increase in the production of cytokines like Tumor Necrosis Factor α (TNF α) and Interleukin-1β, both of which are known to act on bacteria and viruses. An elevated TLR signaling and expression of these cytokines due to prolonged stimulation can worsen hepatic injury in several liver diseases^31^. For example, NASH is known to affect the levels of TLR2 (lipopolysaccharide), TLR4 (peptidoglycan), TLR5 (flagellin), and TLR9 (bacterial DNA), all of which are activated by microbial antigens, thereby leading to inflammatory signaling cascades^31^.

Systemic levels of LPS, a component of Gram-negative bacteria, are higher in cases of liver diseases like NAFLD and NASH. Injecting LPS in mice model for NAFLD enhances liver injury as well as elevates the expression of pro-inflammatory cytokines^32,33^. Wild type mice fed with high fat diet develop steatohepatitis with an increased TLR4 expression and proinflammatory cytokines^32,33^. Further, TLR4 mutants are resistant to LPS induced release of pro-inflammatory cytokines, thus confirming role of TLR4 signaling in NAFLD and NASH^30^. Presence of bacterial DNA (which is higher in NASH patients) leads to elevated expression of TLR9 in NASH models^30^. Experiments with TLR9-deficient models fed with choline-deficient amino acid-defined (CDAA) diet show lesser inflammation, steatosis or fibrosis as compared to those in wild type model^30^. TLR9 signaling affects expression of inflammasome in macrophages, thereby resulting in formation of proinflammatory IL-1β and enhancement of the progression of hepatic injury in NASH^30^.

TLR2 interacts with such Gram-positive bacterial cell wall components like lipoteichoic acids and peptidoglycan. Based on the experimental observations on mice models, insulin resistance induced by high fat diet can be prevented by inhibition of TLR2 signaling^34^. Further, TLR2-deficient mice are seen to be resistant to ‘Choline Deficient Amino Acid’ (CDAA) induced steatohepatitis and have reduced expression of pro-inflammatory cytokines^35^. In contrast, TLR2-deficient mice on ‘Methionine-Choline Deficient’ (MCD) diet display similar or severe steatohepatitis as compared to wild type mice^30^. Although, MCD diet may lead to features of steatohepatitis, it helps in increasing the insulin sensitivity and promotes weight reduction. On the other hand, high fat and CDAA diets lead to weight gain and insulin resistance^36^.

TLR5 binds to bacterial flagellin and plays a protective role for the intestine. TLR5-knockouts develop not only obesity and steatosis, but also display an imbalance in the gut microbiome^30^. Further, transfer of gut microbial communities from TLR5 knockout mice to WT germ-free mice gives rise to metabolic syndrome^30^. Thus, an interplay of gut microbiome and TLR5 probably contribute towards metabolic syndrome pathophysiology.

#### Metabolites transferred from gut through systemic and portal circulation

**Fig. 2:**
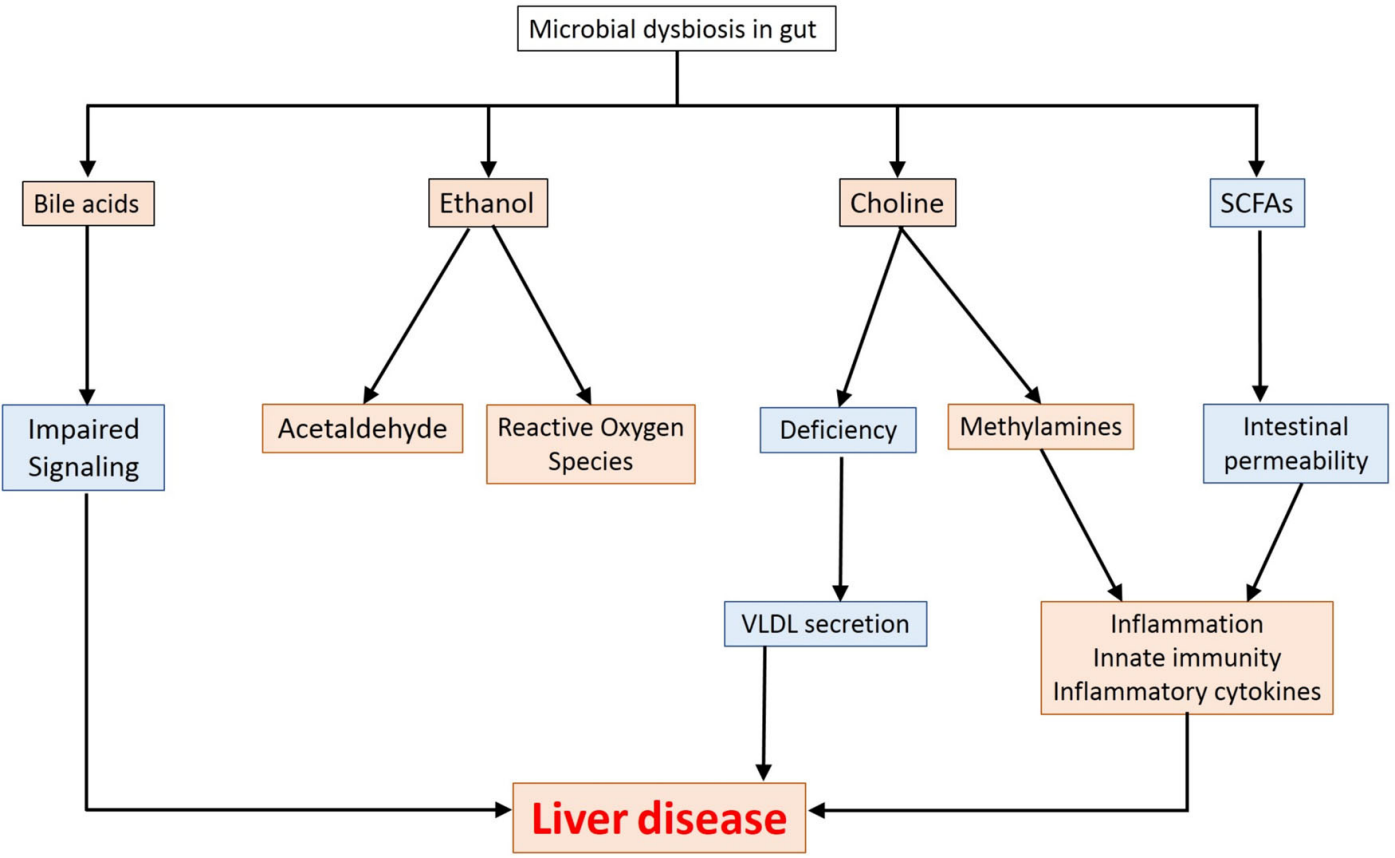
Gut metabolites and liver disease. Metabolites biosynthesized by the microbiome and their impact on onset of liver disease.

Several metabolites biosynthesised in the gut exert multiple effects in liver (depicted in Fig. 2).

##### Trimethylamine and trimethylamine oxide

Choline, a dietary macronutrient, is involved in multiple physiological processes in liver, which include phospholipid biosynthesis (phosphatidyl choline and other membrane lipids), cholesterol metabolism and enterohepatic circulation of bile and lipids^9^. Deficiency of choline in the diet leads to impairment in liver and brain function as well as metabolic processes and muscle movement. Free choline is absorbed by small intestine which then either gets integrated into the membrane or is transferred to liver where it is likely to get converted to betaine, lecithin, etc^37^. Lesser availability of choline leads to accumulation of triglycerides (due to lower formation of phosphatidyl choline by the host) in liver, a factor which has been associated with NASH and also in the manifestation of NAFLD^38^. While a choline-deficient diet induces steatohepatitis, excess choline in diet (exceeding the absorptive capacity of host) moves to the large intestine to get assimilated to Trimethylamine (TMA) by gut microbes (Supplementary File 1–1.1)^39^. Another route for biosynthesis of TMA involves degradation of carnitine obtained from dietary sources like red meat and dairy products (Supplementary File 1–1.1). The TMA thus formed is transferred to liver through portal circulation and gets converted to Trimethylamine Oxide (TMAO), a component which has been implicated in multiple cardiometabolic disorders, hepatic diseases, etc^39^.

##### Short-chain fatty acids

Metabolites like short chain fatty acids (SCFAs) primarily include butyrate, propionate and acetate and are formed in the large intestine as a result of dietary assimilation of polysaccharides, resistant starch, fiber, etc^40^. The SCFAs work as nutrient and energy source for intestinal epithelium and act as precursors for lipogenesis and gluconeogenesis^41^. The butyrate level in the gut helps in maintaining the intestinal integrity as well as permeability^42^. A decrease in butyrate is observed in several liver ailments and alcohol influenced liver injuries^43^.

SCFAs bind and activate G-protein coupled receptors (GPCRs) GPR41 and GPR43^44^. This activation influences peptide-YY secretion as well as causes inhibition of gut motility, thereby increasing the nutrient utilization and yielding of energy. The signaling across GPR41 and GPR43 leads to secretion of GLP1 which in turn reduces the food intake as well as emptying of gastric tract^44^. Further, GPCR signaling also affects regulation of fatty acid oxidation and insulin sensitivity by hepatocytes. Apart from this, GPR43 activation also leads to inhibition of lipolysis and reduced plasma fatty acids^44^.

In addition to GPCR-based signaling, SCFAs can reach the liver through the portal circulation and can have either beneficial or deleterious effects on the liver. For example, increased acetate can be channeled to fatty acid biosynthesis pathway, thereby leading to triglyceride accumulation which has often been correlated to liver ailments^41,45^. Similarly, propionate which acts as a precursor for gluconeogenesis has also been associated to NAFLD^41,46^. On the other hand, butyrate may utilize multiple mechanisms to reduce the pathophysiology associated with liver diseases. For instance, butyrate can activate ‘AMP activated Protein Kinase’ (AMPK), which in turn reduces inflammation and influences glucose as well as lipid metabolism. AMPK further suppresses lipogenic genes^41^. AMPK expression in liver (regulated by butyrate) reduces insulin resistance and obesity. Butyrate can also function as inhibitors of ‘Histone deacetylases’ (HDACs) which can prevent development of liver diseases like NASH and NAFLD at epigenetic level^41^.

Administration of SCFAs has beneficial effects like reduction in hepatic steatosis and insulin resistance^47^. On the contrary, while enrichment of formate and acetate are found in adult subjects at advanced stages of NAFLD, butyrate and propionate are seen to be higher in mild NAFLD^16^. These differences in overall functioning of SCFAs in liver diseases may be affected by factors like diet and environment.

##### Ethanol and acetaldehyde

Ethanol is absorbed mostly in stomach and small intestine via diffusion by gastrointestinal mucosa^48^. Majority of ethanol in large intestine is obtained from systemic circulation. Some of the gut microbes can convert ethanol to acetaldehyde and to lesser extent acetate using alcohol metabolizing enzymes such as alcohol dehydrogenase^49^. Liver also expresses enzymes for ethanol metabolism in response to systemic ethanol content^50^.

Interestingly, while certain small amounts of ethanol are observed in the bloodstream of subjects who do not consume alcohols, pediatric subjects with NASH are seen to possess higher serum ethanol levels as compared to obese children without NASH^51^. These levels of ethanol could be contributed by metabolism by the gut microbiome^52^. Consumption of ethanol is likely to add to the pathophysiology of liver diseases (NASH, NAFLD, etc.) since it may cause not only an increase in intestinal permeability, but also may assist in production of inflammatory cytokines^25^. Endogenous ethanol can increase availability of acetate, a precursor of triglyceride formation through mechanisms involving inhibition of TCA cycle^53^. Ethanol oxidation by CYP2E1 can lead to production of free radicals which is likely to elevate inflammation^54^. Apart from this, ethanol can be metabolized to acetaldehyde which may either disrupt the tight junctions in the intestinal epithelium^55^ or may have oxidant-dependent cytotoxic and metabolic effect on intestinal goblet like cells^56^.

##### Bile aids

Oxidation of cholesterol to form primary bile acids, cholic acid, and chenodeoxycholic acid takes place in the hepatocytes through a multi-step process^57^. These bile acids are further conjugated to glycine or taurine which function as fat emulsifiers in the duodenum for solubilizing fats^57^. The released bile acids enter canaliculi through an export pump and move to the gallbladder where they get stored^58^. The bile acids are released into the duodenum upon consumption of food as a response to increase in production of cholecystokinin^57^. The intestinal microbiome converts these primary bile acids to secondary bile acids such as deoxycholic, lithocholic, and ursodeoxycholic acids^59^.

Chenodeoxycholic acid (CDCA) activates FXR signaling, which in turn helps in not only regulating glucose levels and metabolism (increase insulin sensitivity, glycogen synthesis and inhibit gluconeogenic genes), but also influences cholesterol transport, inhibits lipogenesis and enhances fatty acid oxidation^60^. Bile acids also lead to reduction in expression of lipogenic genes as well as help in reducing triglyceride levels by activating FXR and the pathway involving small heterodimer partner (SHP) and the sterol regulatory element-binding protein 1 (SREBP-1)^61^. FXR also increases the proliferator-activated receptor alpha (PPARalpha) expression which in turn exerts anti-inflammatory effects and regulates lipid as well as glucose metabolism^62^.

While most (95%) of the bile acids are reabsorbed in the distal part of ileum and transported back to liver through portal vein, the remaining gets deconjugated by gut microbes and excreted out in the feces^57^. A small fraction of reabsorbed bile acids is likely to escape uptake into liver and reach the peripheral tissues through systemic circulation. The changes observed in bile acids in liver disease have been detailed in Supplementary File 1–1.2.

### Immune surveillance by liver affects distal organs

Liver plays an important role in immune regulation as well as immunomodulation and possesses almost 80% of all tissue-based macrophages^63^. It affects the innate immunity in other organs and is responsible for secretion of inflammation mediators like serum IL-6 and the acute phase protein CRP^64,65^ in. Thus, it is important to understand the role of gut microbiome, the biosynthesized metabolites in liver and the overall effects on distal organs.

#### Gut–Liver–Brain axis

Hepatic encephalopathy (HE), linking brain function with liver diseases, involves a vast range of neurological and psychiatric abnormalities, ranging from subclinical alterations to coma^11^. HE has often been observed as one of the major complications in individuals with hepatic insufficiency which includes diseases like liver cirrhosis and fibrosis^10^. HE is observed in almost 30–45% of patients with liver cirrhosis and 24–53% of patients with transjugular intrahepatic portosystemic shunt (TIPS)^66^. Having seen the link between the gut and liver, it is important to view this connection with respect to brain pathophysiology as observed in HE, i.e. Gut–Liver–Brain axis

**Fig. 3:**
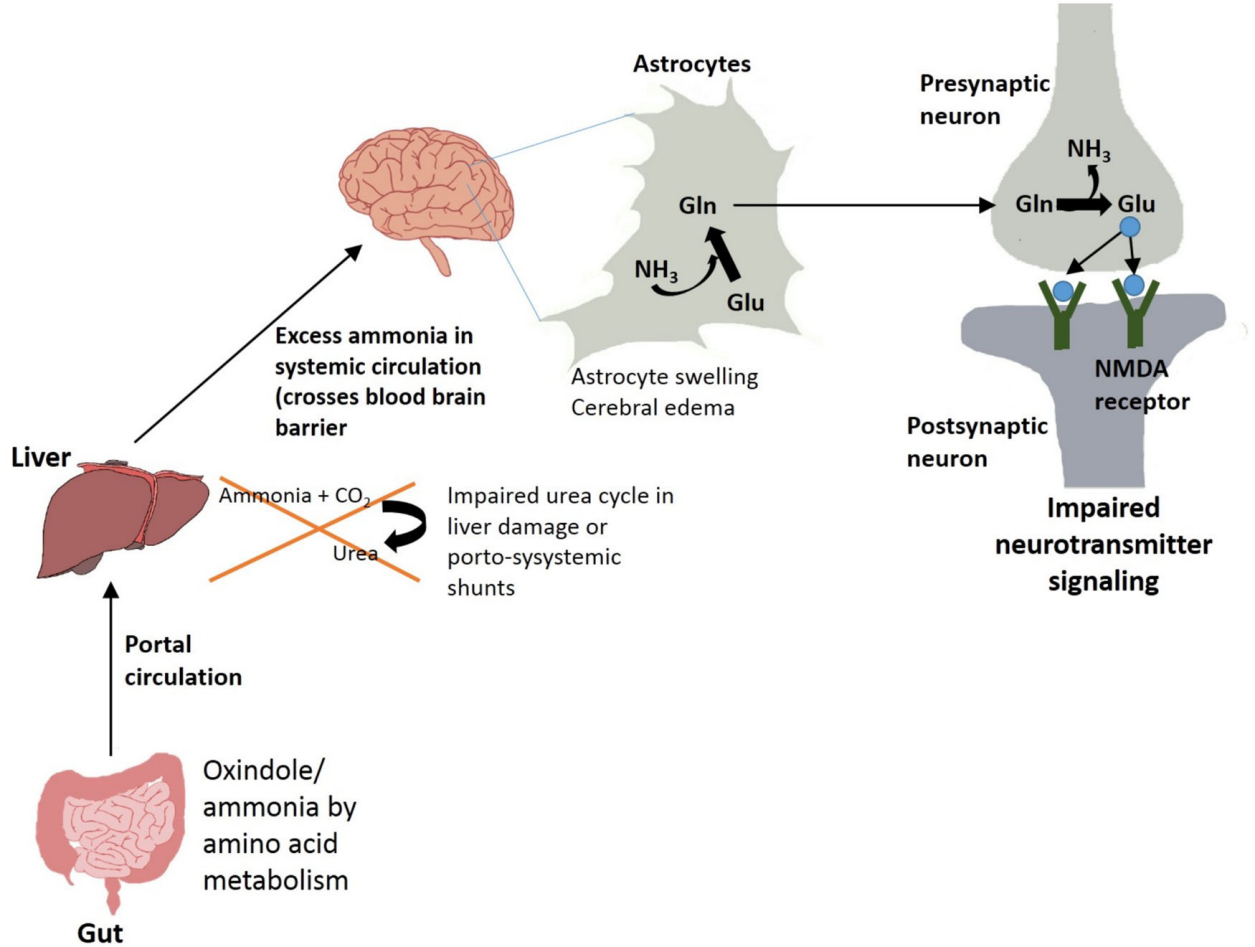
Gut–Liver–brain axis. Impairment of liver urea cycle in liver disease condition leads to increase in uremic toxins and ammonia which reach the brain and affect neurotransmitter signaling and astrocyte swelling.

Gut microbial products like ammonia and oxindole, obtained after metabolism of amino acids, are deleterious for brain^12^ (Fig. 3). Oxindole functions as a sedative by acting as a ligand for voltage operated sodium channels in the brain. Ammonia functions by influencing neurotransmission, pH, membrane potential, astrocyte swelling, etc. Liver diseases like cirrhosis are often associated with insufficiency in detoxification of ammonia and indole derivatives by the liver^10,11^. The reduction in clearance of ammonia from portal vein in cirrhotic patients is accompanied by higher ammonia uptake by brain astrocytes which has been associated to neurological symptoms (Fig. 3). Ammonia is primarily formed in gastrointestinal tract by the action of glutaminase or urease enzymes as well as metabolism of other nitrogen-rich compounds^67^. This ammonia gets into portal circulation and reaches the liver where it is further detoxified by urea cycle. Individuals with portosystemic shunts or liver failure often have compromised liver detoxification abilities which lead to excessive accumulation of nitrogen wastes in systemic circulation (Fig. 3). Excess ammonia is likely to cross blood brain barrier and be absorbed into astrocytes where it possibly gets converted to glutamine^12^. Glutamine thus formed may cause oxidative or osmotic stress and astrocyte swelling, further manifesting as cerebral edema and increased GABAergic activity (Fig. 3)^68^.

Systemic inflammation and sepsis have also been considered as factors which are involved in exacerbation of HE^69^. HE patients often show higher occurrence of inflammatory cytokines like IL-6, IL-18 and TNFα^11^. The presence of systemic and local inflammation has been shown to augment the effect of hyperammonemia in HE. Proinflammatory cytokines can be produced in brain, thereby giving rise to neuroinflammation^70^. Systemic inflammation can occur in case of liver cirrhosis due to multiple factors, one of them being increase in intestinal permeability which can lead to translocation of bacteria and their products into systemic circulation^68^. These bacteria along with their PAMPs help in activating the immune response with release of pro-inflammatory cytokines. The loss in gut barrier integrity in case of liver failure can happen due to factors like reduction in formation of tight junction proteins, reduction in SCFA levels, dysbiosis in gut, endotoxemia, etc.^71^. Systemic inflammation and hyperammonemia may lead to activation of resident macrophages in central nervous system called microglial cells^72^. This may result in formation of brain derived proinflammatory cytokines and result in neuronal death.

#### Gut–Liver–kidney axis

Hepatic failure is often linked to kidney dysfunction or chronic kidney disease (CKD)^73^. A reduction in the estimated glomerular filtration rate (eGFR) of <60 mL/min for more than 3 months is considered as the diagnosis of CKD in cirrhosis. A study in 2019 showed that 46.8% of hospitalized patients with cirrhosis were diagnosed with CKD^74^. Similarly, there is a correlation between loss of kidney function and dyslipidemia^75^. NAFLD leads to lipid accumulation, often involved in aggravating insulin resistance, inflammation, hypertension and obesity, which in turn may influence kidney dysfunction^76^. Increase in biosynthesis of pro-inflammatory, pro-thrombotic factors in NAFLD may contribute towards renal damage^77^. Further, changes in the expression of hepatic lipase lead to high triglyceride levels. Levels of Apolipoprotein B-100 containing lipoproteins biosynthesized in liver also show abnormalities in CKD patients^78^. Systemic inflammation during CKD can cause multiple effects and contribute to NAFLD.

**Fig. 4:**
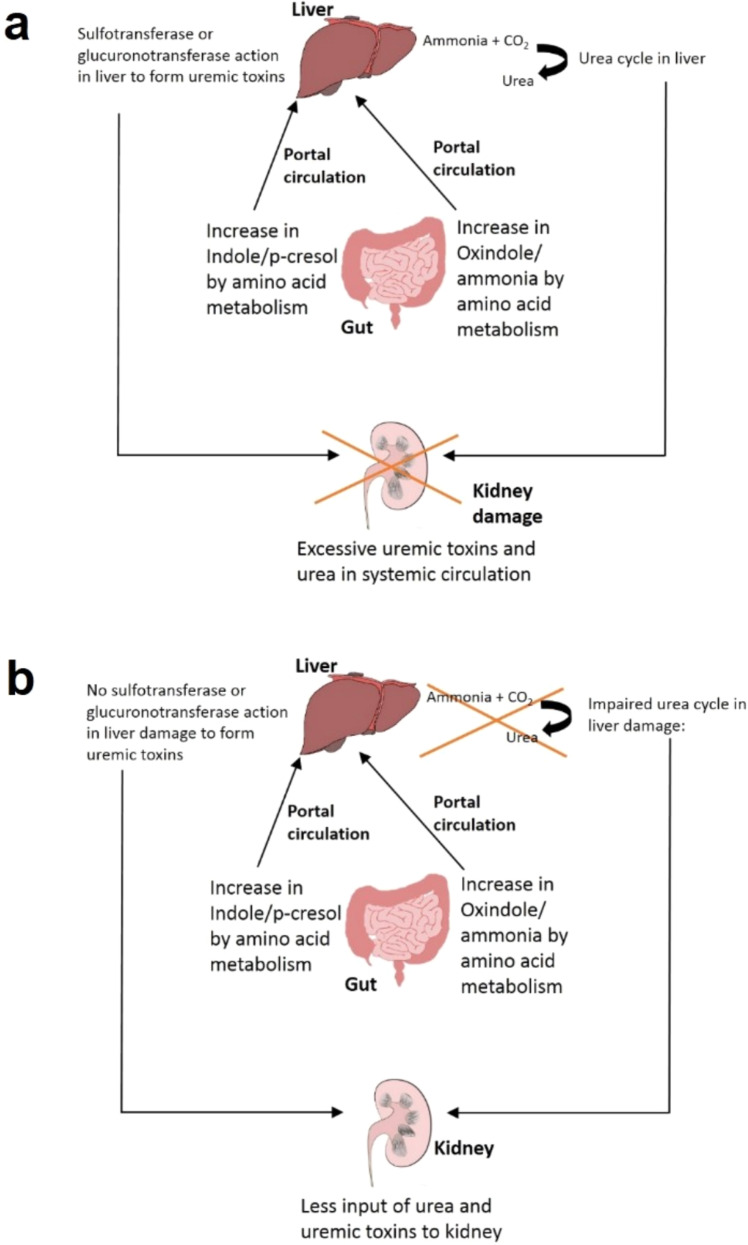
Gut–Liver–kidney axis and liver damage. **a** Gut–Liver–kidney axis without liver damage: Oxindole and cresol produced by gut microbiome are converted to uremic toxins in liver. The uremic toxins reach kidneys through portal circulation. **b** Gut–Liver–Kidney axis with liver damage: Oxindole and cresol produced in the gut are not converted to uremic toxins in liver.

Gut microbiome plays an important role in the connection between liver and kidneys (‘Gut–Liver–Kidney axis’) depicted in Fig. 4a. Dysbiosis in gut microbiome or high level of proteins in diet lead to high protein fermentation in the gut, thereby giving rise to formation of ammonia, indole, p-cresol, etc.^79^ (Fig. 4a). While indole is formed by fermentation of tryptophan by intestinal bacteria, p-cresol is formed by decarboxylation of 4-hydroxyphenylacetic acid which is a product of tyrosine degradation by host enzyme^80,81^. These products are absorbed by intestinal mucosa and taken to the liver where they are further modified by host sulfotransferases or glucoronotransferases to give rise to indoxyl-sulfate, indoxyl glucuronate, p-cresyl-sulfate, and p-cresyl-glucuronate, all of which are uremic toxins^82^. These toxins move into systemic circulation and are cleared from the system by renal filtration. Such toxins also affect the progression of renal ailments and are observed to be elevated in patients with CKD and end stage renal disease (ESRD)^82^ (Fig. 4a). The uremic toxins are expected to act as agonists of aryl hydrocarbon receptor (AhR) and influence release of pro-inflammatory cytokines as well as increase inflammation and oxidative stress^83^. The observed alterations in expression of genes like hepatic cytochrome P450 (CYP) and drug transporter function are expected since these genes have AhR sites on their promoters^84^. This leads to changes in drug metabolism in hepatocytes (Fig. 4a). In patients with CKD and advanced liver disease or cirrhosis, the activity of enzymes responsible for modification in liver (sulfotransferases) is lowered and could contribute towards reduction in the uremic toxin formation^82^ (Fig. 4b). Thus, the amount of uremic toxins in the body in case of kidney damage is also influenced by the liver condition. Further, uremic toxins could have regulatory effects in liver (Fig. 4b).

TMA produced by gut microbiome from choline metabolism is converted to TMAO in liver by flavin-like monooxygenases. The TMAO is carried to kidneys by systemic circulation and cleared by glomerular filtration^85^. TMA and TMAO levels are increased in individuals with renal diseases, CKD, ‘End Stage Renal Disease’ (ESRD), fibrosis, etc^86^. Further, a higher presence of TMAO is associated with liver ailments like NAFLD, NASH, liver cirrhosis, etc^87^. TMAO helps in suppression of bile acid mediated farnesoid X receptor signaling in liver, which in turn leads to aggravation in liver steatosis^87^. This indicates that change in TMAO levels during kidney dysfunction may also influence the physiology of liver.

#### Gut–Liver–Lung axis

An increase in innate immunity concomitant with an increase in inflammatory markers like C-reactive protein (CRP) has been associated with deterioration in lung function and exacerbation of diseases like Chronic Obstructive Pulmonary Disease (COPD)^88^. COPD refers to a lung disease that causes airflow blockage to the lungs and leads to breathing-related problems. The prevalence of steatosis, NASH and fibrosis in COPD patients have been reported to be 41.4%, 36.9% and 61.3%, respectively^89^. The higher innate immune response as well as pro-inflammatory markers like IL-6 have been correlated to pathophysiology of lung ailments^88^. The liver works as a site of immunomodulation with the mevalonate pathway playing a major role. Statins which inhibits the mevalonate pathway in liver is observed to reduce the lung damage^90^. Lovastatin is seen to reduce deleterious effects of macrophage activation in mouse models. Atorvastatin is shown to reduce not only lung inflammatory cells by 30–60%, but also expression of pro-inflammatory genes including reduction in CRP and IL-6^91^.

In addition to mevalonate pathway, liver plays a crucial role in building up an innate immune response in terms of recruitment of macrophages and the neutrophils at the site of lung injury. Studies on mouse model indicate its role in increasing release of IL-6 and acute phase proteins by alveolar macrophages^92^. These proteins are likely to generate chronic inflammation leading to activation of innate immunity in circulation as well as in lungs (termed as innate immune hyper-responsiveness), especially in cases of injury as seen in diseases causing lung damage. Thus, the “Liver–Lung axis” links the innate immune responsiveness and lung injury with the innate immune response regulated by the liver and the mevalonate pathway^88^.

To assess possible link between diet and respiratory diseases, outcomes based on a study on ~120,000 subjects indicate significant reduction in occurrence of COPD with intake of high-fiber diet comprising of whole food grains^93^. Similarly, intake of whole grains shows substantial improvements in FEV1 (Forced Expiratory Volume in 1 s) of smokers (200 ml change across diet quartiles) as compared to non-smokers (50 ml change across dietary quartiles)^94^. Evidences indicate the effect of dietary fibers on immunomodulation of innate immune response. Comparison of effects of dietary fiber intake on specific causes of death suggest dietary fiber’s role in reduction in mortality in individuals suffering from respiratory ailments^95^. Similarly, 50–60% reduction in mortality due to intake of high-fiber diet comprising of whole grains (HR = 0.47 for men and 0.40 for women) has been observed in a European study on >450,000 individuals^96^. A study on a cohort of 35,339 Swedish women showed that a long-term intake of dietary fiber could be associated with a 30% lower risk of COPD^97^. Epidemiological and clinical studies also suggest role of high-fiber diets in reducing systemic inflammation and leading to decrease markers of inflammation like CRP and IL-6^98^.

Looking into the ‘Gut–Liver–Lung axis’, one of the possible ways by which dietary fiber provides benefit is by stimulating the growth of beneficial bacteria in gut which help in biosynthesis of SCFAs through fermentation^3^. Some SCFAs get absorbed and enter portal circulation, thereby affecting organs like liver^99^. SCFAs function by modulating innate immune activation. High-fiber diet is seen to reduce pulmonary inflammation in murine models. SCFAs influence the migration of neutrophils and macrophages via GPCR activation which helps in reducing pulmonary inflammatory response^44^. Furthermore, SCFAs inhibit the HMG-CoA reductase which catalyzes the rate limiting step of mevalonate pathway^41^. This inhibition enables reduction in inflammatory markers and lowering of the innate immune response. HMG-CoA reductase inhibition in the liver ‘dampens’ the innate immune response and lowers serum levels of IL-6 through the IL-6 trans-signaling pathway^88^. This leads to a downstream inhibitory effect on the pro-inflammatory transcription factors NF-ĸB and signal transducer and activator of transcription^88^.

#### Gut–Liver–Heart axis

Liver diseases like NAFLD, NASH, and cirrhosis, which have been associated with changes in gut microbiome, also correlate with the occurrence cardiovascular (CV) ailments comprising disorders of heart and blood vessels (‘Gut–Liver–Heart axis’)^100^. Non-alcoholic fatty liver disease (NAFLD) is associated with a higher risk of cardiovascular disease (CVD) which includes coronary heart disease (CHD), heart failure, stroke, and arrhythmia^101^. A follow up study on 285 individuals with biopsy proven NAFLD and no incidence of CVD showed an occurrence of a cardiovascular event in 9.1% of these advanced stage individuals within 5 years of follow up^102^. Exposure to lipopolysaccharide and its binding to TLR4 lead to an inflammatory immune response with release of pro-inflammatory cytokines^103^. This process promotes LDL oxidation, formation of atherosclerotic plaques and thrombogenesis^104^.

Dietary intake which are higher in choline, betaine or carnitine (e.g. red meat) leads to formation of TMA by gut bacteria, which further gets converted to TMAO in liver^39,87,105^. The increase in TMAO levels is associated to liver as well as CV events^86^. Thus, TMAO can be considered as marker for a deteriorating liver condition or Cardiovascular health^86^. TMAO concentrations have been related to atherosclerosis which is one of the major causes of CVDs as well as ‘Major adverse cardiovascular events’ (MACE) including myocardial infarction, stroke, etc.^86^. Higher TMAO levels and expression of pro-inflammatory cytokines (TNF-α and IL-1β) are observed to be accompanied with cardiac dysfunction in mouse models^106^. The inhibition of choline TMA lyase enzyme by chemicals like 3,3-dimethyl-1-butanol (DMB) can prevent increase in TMA levels as well as other outcomes^107^.

There exists a link between endothelial dysfunction and TMAO levels. TMAO treatment carried out on human monocytic THP-1 cells and human umbilical vein endothelial cells (HUVEC) reveal an increase in monocyte adhesion which lead to increased expression of VCAM-1^108^. Additionally, lipid metabolism is also regulated by TMAO which alters cholesterol and sterol metabolism^106^. Catabolism of cholesterol involves bile acid synthesizing enzyme Cyp7a1 catalyzing the rate limiting step. TMAO lowers the expression of this enzyme which has been associated with atherosclerosis^109^. Supplementation of choline, carnitine or TMAO may decrease reverse cholesterol transport. Further, TMAO influences the increase in expression of CD36 and SR-A1 (scavenger receptors) which leads to lipid accumulation and foam cell formation^106,110^. These effects are induced by oxidative modification of LDL in presence of TMAO. The inhibition of MAPK by inhibitors leads to a reduction in expression of CD36 as well as foam cell formation, indicating action of MAPK/JNK pathway in atherosclerosis induced by TMAO^111^.

Some inconsistencies regarding link of plasma TMAO levels and CVDs still exist. For example, short- and long-term higher plasma TMAO levels are observed in people after bariatric surgery. The result is unexpected as high TMAO concentrations increase the CVD risk while the aim of bariatric surgery is to reduce CVD risk^39^. However, recording their diet and gut microbiome could have thrown some light on whether TMAO levels were found to be higher in subjects as a result of a surgery-induced change in gut microbiome or due to a greater ingestion of carnitine (a TMA precursor) which is often promoted as a weight loss inducing supplement.

There are also certain contrasting observations regarding role of TMAO in CVDs. Although, TMAO is shown to correlate with inflammatory markers and endothelial dysfunction, some studies indicate such associations only in case of HIV and type-II diabetes^39^. Few studies also indicate no significant correlation of TMAO levels with inflammatory marker CRP. Protective role of TMAO in CVDs have also been reported. After carnitine supplementation, improvement is seen in some CVDs despite an increase in TMAO and TMA^112^. Food items like marine fish contain high levels of TMAO which are observed in circulation after dietary intake. Despite that, studies on mice show that supplementation of fish oil along with a high fat diet alleviate damage caused by TMAO including increased glucose tolerance and inflammation of adipose tissue^113^.

In summary, the Gut–Liver axis refers to bidirectional communication between gut, its microbiome and the liver. The metabolites produced by gut microbiome are connected with liver through systemic circulation, portal circulation and the bile duct. While the metabolites produced in the gut influence immunity, metabolism and bile acid production, the bile acids produced in liver in turn regulate the gut microbial composition as well as gut epithelial barrier integrity. Therefore, a dysbiosis in gut microbiome not only leads to a change in the bile acid pool within the host, but also often been observed in liver related pathophysiologies like NAFLD, NASH, ALD, etc. Further, since some gut bacteria are capable of metabolizing bile acid, the bile acid pool determines and influences the composition of gut microbiome. The shifting level of bile acids impacts the intestinal integrity and metabolism by affecting FXR signaling. Exposure of liver immune cells to metabolites like TMAO produced by gut bacteria can increase liver inflammation. Further, the liver regulates the innate immunity as well as metabolism of various toxins and metabolites in other organs. In other words, a deterioration in liver condition can also impact the metabolism signaling and immunity in other important host organs. Hence, the Gut–Liver axis can be extended to distal organs like Gut–Liver–Brain, Gut–Liver–Kidney, Gut–Liver-Heart and Gut-Liver-Lung axes.

Findings from the Gut-Liver-X (X being Brain or Kidney or Heart or Lung) axes indicate potential of utilizing gut microbiome as diagnostic and therapeutic strategy for early detection and management of not only liver diseases, but also diseases effecting other organs (e.g., chronic kidney disease, hepatic encephalopathy, cardiovascular ailments, respiratory obstructions, etc.). Identifying microbiome signatures which can be indicative of different health conditions is an active area of research. An understanding of Gut–Liver axis and interactions with distal organs can further help in identifying probiotic and fecal transplant strategies as preventive therapeutic regimes for liver ailments. Although certain studies have indicated potential use of probiotics as a therapy for chronic liver diseases, long-term impacts as well as effects on host-microbiome balance have yet to be elucidated (Supplementary Table 1). Clinical trials with standardized dosage of probiotics and extended duration of administration along with regular follow-ups are necessary to confirm the efficacy of the probiotics in manipulating the Gut–Liver axis as well as understanding their impacts on other organs like brain, kidney, lung and heart.

## Supplementary information

Supplementary Material

## Data Availability

All required data has been provided within the manuscript.
