# Supplementary material for: Host-microbiome interactions: Gut-Liver axis and its connection with other organs
Source: NPJ Biofilms Microbiomes. 2022 Nov 1;8:89. doi: 10.1038/s41522-022-00352-6 (PMC9626460; doi:10.1038/s41522-022-00352-6)
Supplement: Supplementary file 1 — Supplementary Material [file 41522_2022_352_MOESM1_ESM.pdf]

1.1 Conversion of choline and carnitine to TMA

One way of conversion of choline to TMA (with a concomitant release of acetaldehyde) involves the action of bacterial choline utilization gene cluster (cut) comprising of a glycyl radical enzyme choline TMA (CutC) lyase capable of initiating choline degradation. Choline and carnitine can also be converted to TMA by another pathway, wherein they are first converted to betaine by host as well as by certain gut bacteria, which then acts as an electron acceptor in an oxidation-reduction reaction to yield TMA (with concomitant release of acetate)[7,8].

About half of the dietary carnitine is absorbed by the intestine, the 3-hydroperoxybutyryl moiety of the remaining half is utilized by gut microbiome and metabolized to yield TMAO. In this process, the two component Rieske type oxygenase/reductase (cntAB) catalyseses the conversion of carnitine to TMAO, which is accompanied by an electron transfer [9].

1.2 Biotransformation of bile acids by gut microbiome

Biotransformation of bile acids by gut bacteria involves deconjugation, dehydroxylation, oxidation and desulfation [10]. Bacteria belonging to the genera such as Bacteroides, Lactobacillus, Bifidobacterium, Clostridium and Listeria utilize their bile acid hydrolases to deconjugate taurine and glycine groups from primary bile acids [10]. The deconjugated primary bile acids include cholic acid (CA) and chenodeoxycholic acid (CDCA). Another set of gut microbes (e.g., belonging to Eubacterium and Clostridium) then come into play and using their 7α-dehydroxylases convert these deconjugated primary bile acids into secondary bile acids like deoxycholic acid (DCA), lithocholic acid (LCA) and ursodeoxycholic acid (UDCA) [10]. Apart from this, hydroxysteroid dehydrogenases from multiple gut bacteria oxidize several toxic bile acids to less toxic UDCA. Gut bacteria also produce sulfatases which lead to desulfation of bile acids, thereby facilitating the reabsorption as well as maintenance of bile acid pool. While deconjugated primary bile acids and secondary bile acids like DCA are known to have better antibacterial properties than CA, unconjugated bile acids show antibacterial action by disruption of bacterial membranes leakage of cellular contents[10].

REFERENCES

1. Tripathi, A. et al. The gut-liver axis and the intersection with the microbiome. Nat. Rev. Gastroenterol. Hepatol. 15, 397–411 (2018).

2. Wiedeman, A. M. et al. Dietary Choline Intake: Current State of Knowledge Across the Life Cycle. Nutrients 10, (2018).
3. Zhu, Y. et al. Carnitine metabolism to trimethylamine by an unusual Rieske-type oxygenase from human microbiota. Proc. Natl. Acad. Sci. U. S. A. 111, 4268–4273 (2014).
4. Wang, C. et al. Role of Bile Acids in Dysbiosis and Treatment of Nonalcoholic Fatty Liver Disease. Mediators of Inflammation vol. 2019 e7659509 https://www.hindawi.com/journals/mi/2019/7659509/ (2019).
5. Prawitt, J., Caron, S. & Staels, B. Bile acid metabolism and the pathogenesis of type 2 diabetes. Curr. Diab. Rep. 11, 160–166 (2011).
6. Watanabe, M. et al. Bile acids lower triglyceride levels via a pathway involving FXR, SHP, and SREBP-1c. J. Clin. Invest. 113, 1408–1418 (2004).
7. Pineda Torra, I. et al. Bile acids induce the expression of the human peroxisome proliferator-activated receptor alpha gene via activation of the farnesoid X receptor. Mol. Endocrinol. Baltim. Md 17, 259–272 (2003).

Disease	Primary findings	Study organism/design	Reference Pubmed ID
NASH	Probiotic-treatment reduces<br>- serum ALT and decreases liver stiffness indicating improvement of the inflammation state<br>- reduction in serum cholesterol	NASH patients<br><br>- Control group: fed low-fat & low-calorie diet<br>- Test group: fed low-fat & low-calorie diet + probiotic + prebiotic. Administered prebiotic fructooligosaccharides and capsule containing probiotics L. casei, L. rhamnosus, L. bulgaris, Bifidobacterium longum, and Streptococcus	PMID: 29148175
NASH (Methionine-choline deficient diet induced)	Intake of known probiotic L. casei leads to decrease in<br>- Methionine-choline deficient (MCD)-diet-induced NASH development<br>- serum lipopolysaccharide<br>- inflammation in liver and colon<br>- liver fibrosis	Methionine-choline deficient (MCD) diet induced NASH mice model<br><br>Control group 1: fed chow diet (NCD),<br>Control group 2: fed MCD diet<br>Test group: fed MCD diet plus daily oral administration of L. casei	PMID: 24113768
NASH (High fat diet induced)	L. paracasei intake significantly lowers<br>- hepatic fat deposition<br>- serum ALT level<br>- TLR4 expression<br>- NADPH oxidase-4<br>- TNF-α, interleukin 4 (IL-4)<br>- peroxisome proliferator activated receptor gamma (PPAR-γ)<br>- PPAR-δ	High-fat induced NASH mice model<br><br>Control group: fed standard chow + 10 % fructose diet<br>Test group: fed L. paracasei	PMID: 26143342
NAFLD	Treatment with probiotics Lactobacillus bulgaricus and Streptococcus thermophilus decreases<br>- alanine amino transferase<br>- aspartate aminotransferase activity<br>- gammaglutamine transferase levels	NAFLD patients<br><br>Control group: Placebo treated<br>Test group: treated with one tablet per day with 500 million of Lactobacillus bulgaricus and Streptococcus thermophilus	PMID: 22013734
NAFLD	Probiotic intake significantly decreases<br>- insulin resistance<br>- TNF-α<br>- IL-6	NAFLD patients<br><br>Control group: Placebo-treated<br>Test group: consumed 2 capsules/day probiotic Lactocare with 7 strains: Lactobacillus casei 3 £ 109 CFU/g, Lactobacillus acidophilus 3 £ 1010 CFU/ g, Lactobacillus rhamnosus 7 £ 109 CFU/g, Lactobacillus bulgaricus 5	PMID: 26430826

		£ 108 CFU/g, Bifidobacterium breve 2 £ 1010 CFU/<br>g, Bifidobacterium longum 1 £ 109 CFU/g, and Streptococcus thermophilus 3 £ 108 CFU/g	
NAFLD	Probiotic-treated patients show improvement in<br>- hepatocyte ballooning<br>- lobular inflammation<br>- NAFLD activity score (NAS)<br>- ALT levels<br>- Tumor necrosis factor-α<br>- endotoxins	NAFLD patients<br><br>Control group: Placebo-treated<br>Test group: consumed multi-strain Probiotic comprising lactic acid bacteria and bifidobacteria: Lactobacillus paracasei DSM 24733, Lactobacillus plantarum DSM 24730, Lactobacillus acidophilus DSM 24735, Lactobacillus delbrueckii subsp. bulgaricus DSM 24734, Bifidobacterium longum DSM 24736, Bifidobacterium infantis DSM 24737, Bifidobacterium breve DSM 24732, and Streptococcus thermophilus DSM 24731	PMID: 31423319
NAFLD	Probiotic consumption improved<br>- hepatic enzymes<br>- serum total cholesterol<br>- low-density lipoprotein cholesterol levels	NAFLD patients<br><br>Control group: consumed conventional yogurt<br>Test group: consumed 300 g/d of probiotic yogurt containing Lactobacillus acidophilus La5 and Bifidobacterium lactis Bb12	PMID: 25306266
NAFLD	Consumption of synbiotic significantly reduces<br>- serum levels of fasting blood sugar<br>- TAG<br>- most of the inflammatory mediators	NAFLD patients<br><br>Control group: placebo-treated<br>Test group: consumed synbiotic supplement with seven strains (Lactobacillus casei, Lactobacillus rhamnosus, Streptococcus thermophilus, Bifidobacterium breve, Lactobacillus acidophilus, Bifidobacterium longum and Lactobacillus bulgaricus) along with prebiotic fructo-oligosaccharide)	PMID: 28345499
NAFLD	Intake of symbiotic combination of prebiotics and probiotic alters the fecal microbiome and does not reduce liver fat content or markers of liver fibrosis	NAFLD patients<br><br>Control group: intake of placebo<br>Test group: intake of symbiotic combination of prebiotic fructo-	PMID: 29787859

		oligosaccharides and probiotic Bifidobacterium animalis subsp. lactis BB-12	
NAFLD	Decrease in<br>- alanine aminotransferase and aspartate aminotransferase<br>- mean cholesterol<br>- low-density lipoprotein-C<br>- triglycerides	Obese children with sonographic NAFLD<br><br>Control group: intake of placebo<br>Test group: intake of Probiotic capsule containing Lactobacillus acidophilus ATCC B3208, Bifidobacterium lactis DSMZ 32269, Bifidobacterium bifidum ATCC SD6576 and Lactobacillus rhamnosus DSMZ 21690	PMID: 28230607
NAFLD (Fructose induced steatosis)	Consumption of known probiotic L. casei in diet prevents onset of fructose-induced NAFLD by influencing the TLR-4-signalling in the liver	C57BL/6J mouse model of fructose-induced steatosis<br><br>Test group: fed with fructose solution + L.casei<br>Test group 3: fed with tap water + L.casei	PMID: 22749137
NAFLD (High fat diet induced)	Consumption of L. plantarum<br>- restores liver function<br>- reduces proinflammatory cytokines<br>- decreases expression of lipid metabolism in the liver	Rats with high fat diet induced NAFLD<br><br>Control group 1: fed normal diet<br>Control group 2: fed high fat diet (HFD)<br>Test group: fed HFD plus L. plantarum NCU116	PMID: 25317840
NAFLD (High fat diet induced)	Intake of cholesterol-lowering probiotics leads to<br>- up-regulating CYP7A1, LDL-R, FXR mRNA and PPAR-α protein<br>- down-regulating the expression of HMGCR, PPAR-γ and SREBP-1c.	Sprague–Dawley rats with high fat diet induced NAFLD<br><br>Control group 1: fed normal diet<br>Control group 2: fed High Fat diet (HFD)<br>Test group 1: fed HFD containing anthraquinone from Cassia obtusifolia L. (AC)<br>Test group 2: fed HFD containing cholesterol-lowering probiotics (P)<br>Test group 3: fed HFD containing AC and P	PMID: 26375281
NAFLD (High-fact induced)	Administrated of Lactobacillus rhamnosus GG lowers<br>- expression levels of genes involved in cholesterol synthesis<br>- suppression of FXR and FGF15 signaling	High-fat diet (HFD) induced NAFLD mice model<br><br>Control group: fed normal diet<br>Test group: treated with Lactobacillus rhamnosus GG	PMID: 27018382

	- upregulation of hepatic CYP7A1		
NAFLD (choline-deficient/L-amino acid-defined diet induced)	Treatment with MIYAIRI 588<br>(a pharmaceutical product of C. butyricum)<br>- reduces hepatic lipid deposition<br>- significantly improves triglyceride content, insulin resistance, serum endotoxin levels, and hepatic inflammatory indexes	Choline-deficient/L-amino acid-defined diet-induced NAFLD rat models<br><br>Control group 1: fed low fat diet<br>Control group 1: fed high fat diet (HFD)<br>Test group: fed HFD coated with CBM588 spores	PMID: 24166662
Cirrhosis and Hepatic encephalopathy	Intake of probiotic preparation VSL#3<br>-reduces hospitalization risk for patients with Hepatic encephalopathy and cirrhosis<br>-end-stage liver disease scores improved	Hepatic encephalopathy and Cirrhosis patients recovered from overt HE and the liver<br>disease had been stable for at least 4 weeks<br><br>Control group: placebo treated<br>Test group: administered probiotic VSL#3	PMID: 25450083
NAFLD [High Sucrose High Fat (HSHF) diet induced]	Probiotic supplement<br><br>- Improves liver pathology<br>- Reduces in serum LPS<br>- Downregulates liver TLR4	HSHF-diet induced NAFLD rat models<br><br>Control group: fed HSHF diet<br>Test group: fed probiotic supplement consisting of 0.5 × 106 colony-forming units (CFU) live Bifidobacterium infantis and Lactobacillus acidopilus and<br>0.5 × 105 CFU live Bacillus cereus	PMID: 28349964
Hepatic steatosis (High fat diet induced)	Kefir supplementation<br>- Improves histopathological liver lesion<br>- up-regulates fatty acid oxidation genes, PPARα, in both the liver and adipose tissue<br>- Reduces the plasma concentration of IL-6, a proinflammatory marker	High fat diet induced hepatic steatosis in C57BL/6 mice model<br><br>Control group: fed milk<br>Test group: fed kefir for 12 weeks	PMID: 28384519

Supplementary Table 1: Role of probiotics in liver disease
